# Hybrid Nanocarriers for Cancer Therapy: Advancements in Co-Delivery of Gene Therapy and Immunotherapy

**DOI:** 10.3390/ijms27010248

**Published:** 2025-12-25

**Authors:** Kulzhan Berikkhanova, Isah Inuwa, Abdulrahman Garba Jibo, Nurzhan Berikkhanov, Nurzhan Bikhanov, Yessenkhan Sultan, Ardak Omarbekov

**Affiliations:** 1 National Laboratory Astana, Nazarbayev University, Astana 010000, Kazakhstan; 2 University Medical Center, Nazarbayev University, Astana 010000, Kazakhstan; 3 Scientific and Educational Center of Surgery, Astana Medical University, Astana 010000, Kazakhstan; 4 School of Medicine, Nazarbayev University, Astana 010000, Kazakhstan; 5 Department of Industrial Pharmacy, Faculty of Pharmaceutical Sciences, Chulalongkorn University, Bangkok 10330, Thailand; 6 Center of Surgery, City Polyclinic № 2, Taraz 080000, Kazakhstan; 7 Medical Center LLP “Sultan-Medicus”, Almaty 050000, Kazakhstan

**Keywords:** cancer, gene therapy, hybrid nanocarriers, immunotherapy, precision oncology

## Abstract

Over the years, cancer has continued to be a leading global health threat, prompting researchers to explore advanced therapies that go beyond traditional treatments like chemotherapy and radiotherapy. Among these advanced therapies, gene therapy and immunotherapy have shown significant promise in treating cancer by either altering genetic makeup or stimulating the immune system. However, their clinical applications face significant obstacles such as poor drug delivery, rapid degradation, and immune system clearance. Hybrid nanocarriers have emerged as a transformative development in modern precision oncology, enabling the co-delivery of gene therapy and immunotherapy agents in a highly targeted manner to address the persistent limitations of traditional cancer treatments. This review focuses on hybrid nanocarrier systems specifically engineered for co-delivery applications and critically evaluates when and how these multifunctional platforms outperform conventional single-modality or non-hybrid formulations. We compare key hybrid architectures in terms of payload compatibility, pharmacokinetics, immune modulation, and translational readiness, and examine the influence of tumor microenvironmental characteristics on their therapeutic performance. Particular emphasis is placed on stimuli-responsive designs, biomimetic surface engineering, and artificial intelligence–assisted optimization as emerging strategies to enhance co-delivery efficacy. By synthesizing current evidence and identifying key scientific and manufacturing gaps, this review aims to provide a practical foundation for advancing hybrid nanocarriers from laboratory development to clinically meaningful, personalized cancer therapies.

## 1. Introduction

Cancer remains one of the top causes of death worldwide, with millions of new diagnoses every year. Global cancer statistics for 2022, as reported by the International Agency for Research on Cancer (IARC), show approximately 20 million new cancer cases globally, including nonmelanoma skin cancers (NMSCs), and 9.7 million cancer-related deaths [1]. Although chemotherapy and radiotherapy have long been cornerstones of cancer treatment, these approaches come with significant limitations. Their tendency to affect both healthy and malignant cells usually results in severe side effects and the development of drug resistance [2].

To address these challenges, cancer therapy has shifted toward more personalized strategies, particularly gene therapy and immunotherapy. Gene therapy targets the underlying genetic faults driving cancer progression, while immunotherapy empowers the body’s own immune system to identify and eliminate malignant cells [3,4]. Yet, despite their promise, these advanced therapies face substantial practical hurdles. Their effectiveness is frequently hampered by poor cellular uptake, rapid degradation in the body, and immune clearance before the drugs reach tumor sites.

Overcoming these delivery obstacles, researchers have developed hybrid nanocarriers, a complex delivery system that merges the advantages of various materials such as lipids, polymers, and inorganic compounds [5]. These nanocarriers protect therapeutic agents and guide them precisely to their targets, thereby minimizing collateral damage to healthy tissues. Notably, the co-delivery of gene therapy and immunotherapy within a single hybrid nanocarrier has emerged as a particularly promising strategy, offering the potential for synergistic antitumor effects, enhanced therapeutic precision, and improved safety profiles [6]. Despite these advances, several critical questions remain unresolved.

How can hybrid nanocarriers be optimally engineered to navigate the complex and heterogeneous tumor microenvironment (TME)? How can immune-related adverse effects be mitigated while preserving therapeutic efficacy? Addressing these issues underscores the need for continued innovation and systematic evaluation of co-delivery platforms.

Recent reviews have discussed hybrid nanocarriers as platforms for chemotherapy or general cancer drug delivery, and others have summarized gene or immunotherapy strategies separately [5,7,8]. However, a focused and critical analysis of hybrid nanocarriers specifically engineered for the co-delivery of gene therapy and immunotherapy, integrated with a detailed consideration of tumor microenvironmental barriers and translational constraints, remains limited. In this context, the present review has three main objectives: (i) to systematically categorize the principal classes of hybrid nanocarriers used for gene–immunotherapy co-delivery and compare their design trade-offs in terms of payload capacity, pharmacokinetics, and safety; (ii) to analyze how TME heterogeneity, immune suppression, and extracellular matrix remodeling shape the performance and clinical suitability of these systems; and (iii) to highlight design principles and selection criteria that indicate when the additional complexity of hybrid carriers is justified over simpler nanoformulations for specific tumor and TME contexts.

Accordingly, this review highlights recent advances in the design and functionality of hybrid nanocarriers for the co-delivery of gene- and immune-based cancer therapies. We discuss how emerging strategies, including biomimetic surface coatings, stimuli-responsive release mechanisms, and artificial intelligence–guided optimization, can be leveraged to improve co-delivery performance and overcome tumor microenvironment–associated resistance.

By synthesizing current preclinical and early clinical data and explicitly outlining the remaining scientific, safety, and manufacturing gaps, this review aims to provide clear and prudent foresight for the rational development and clinical translation of hybrid nanocarriers in precision oncology.

## 2. Types of Nanoparticles and Their Roles in Hybrid Nanocarriers

Hybrid nanocarriers are created by combining two or more classes of nanomaterials to overcome the individual limitations of single-component systems. To understand why hybrid platforms are increasingly favored in co-delivery of gene therapy and immunotherapy, it is essential to outline the major nanoparticle classes they are built from, including their structural characteristics, advantages, and constraints. Recent advances in nanotechnology have expanded the functional diversity of lipid-based, polymer-based, inorganic, and biomimetic nanoparticles, making them core components in next-generation hybrid systems for cancer therapy.

### 2.1. Lipid-Based Nanoparticles

Lipid-based nanoparticles are organized from phospholipids, cholesterol, and ionizable lipids, forming bilayers or lipidic cores that resemble biological membranes [9]. Their amphiphilic nature enables hydrophobic drugs to embed within lipid layers and nucleic acids to localize within aqueous compartments, giving them dual-cargo versatility [10]. A major advantage of modern ionizable LNPs is their efficient endosomal escape, which is driven by pH-dependent protonation of lipids that destabilize endosomal membranes [11]. These same ionizable lipids also reduce systemic toxicity due to their neutral charge at physiological pH [12].

However, lipid systems suffer from rapid clearance by the liver and spleen, requiring PEGylation or membrane coating to improve circulation time [13]. Storage instability and sensitivity to oxidation also limit their broad use [14]. A notable example is the use of lipid nanoparticles (LNPs) for in vivo delivery of CRISPR–Cas9 components, which has enabled efficient gene editing in hepatocytes with minimal toxicity. LNPs facilitate the delivery of Cas9 mRNA and guide RNA to the liver, resulting in successful genome editing and therapeutic outcomes in animal models [15].

### 2.2. Polymer-Based Nanoparticles

Polymeric nanoparticles consist of a solid matrix formed from polymers such as Poly (lactic-co-glycolic acid) (PLGA), Poly-β-amino esters (PBAEs), Polyethylene Glycol (PEG), chitosan, or dendrimers, creating stable structures capable of controlled degradation [16,17,18]. PLGA is a biodegradable polymeric nanoparticle. It was approved by the FDA and is widely used in drug delivery because of its biocompatibility, ability to solubilize a wide variety of drugs, and tunable degradation. PLGA nanoparticles degrade into lactic and glycolic acid, enabling sustained release of immunotherapeutic and gene-delivery agents [19].

Their tunable architecture enables precise control of surface charge, degradation rate, and functional ligand attachment, making them suitable for long-term release of genetic and immune therapeutics [20]. An advantage of polymeric systems is their structural versatility: cationic polymers such as Poly-β-amino esters (PBAEs) enable efficient electrostatic loading of DNA and RNA, while biodegradable variants reduce long-term toxicity [21].

Limitations include cytotoxicity associated with high cationic charge density, polymer heterogeneity, and unpredictable drug leakage in vivo [22]. Compared to lipid-based nanoparticles, polymeric nanoparticle formulations are more likely to induce an immune response due to the high molecular weight of the polymers used [23].

One recent example of polymeric nanoparticles in drug delivery is a poly (β-amino ester) (pBAE) system, which is engineered to co-deliver doxorubicin and BCL-2 siRNA to overcome multidrug resistance in lung cancer. This system utilizes a dual pH- and redox-responsive release mechanism, ensuring precise tumor targeting and minimizing leakage in the circulation, thereby enhancing the therapeutic efficacy of doxorubicin in resistant cancer cells [24].

### 2.3. Inorganic Nanoparticles

Inorganic nanoparticles, including metals, semiconductor materials, and metal oxides, are extensively studied in the biomedical field due to their controllable size, unique physical properties, and biocompatibility [25]. These nanoparticles exhibit characteristics such as optical properties, electrical conductivity, magnetic properties, and catalytic activity, making them promising candidates for therapeutic applications in disease treatment [25]. Specifically, materials like gold, silica, manganese dioxide, hafnium oxide, and iron oxide demonstrate rigid crystalline or porous structures that allow for precise shape and size control [16].

Gold nanorods (AuNRs) are widely used as photothermal agents because their longitudinal surface-plasmon resonance can be tuned into the near-infrared (NIR) window, where they efficiently convert NIR photons into heat and can also promote intracellular drug uptake [26]. Manganese-oxide (MnO_2_) nanomaterials act as catalytic “nano-enzymes” that decompose tumor-associated H_2_O_2_ into O_2_, thereby alleviating hypoxia; the released Mn^2+^ can directly engage the cGAS–STING pathway, enhancing innate immune activation [27].

Despite their therapeutic promise, inorganic nanoparticle carriers face significant challenges for clinical translation, including slow degradation, potential long-term accumulation in organs, and metal-associated toxicity, which can limit their safety profile in vivo [28].

### 2.4. Biomimetic and Cell-Derived Nanoparticles

Biomimetic nanoparticles leverage cell membrane components to exploit natural recognition pathways and evade immune clearance. This structural mimicry enables immune evasion and enhances targeted interactions with tumor or immune cells [29,30]. Unlike conventional synthetic nanoparticles, these systems harness the unique properties of biological membranes and structures, allowing for improved tumor targeting and immune system interaction. By mimicking natural cellular behavior, these nanoparticles can achieve higher precision in drug delivery and immunotherapy [30]. Among the most promising innovations are cell membrane-coated nanoparticles, exosomes, and bacterial outer membrane vesicles (OMVs) [31].

Cell membrane-coated nanoparticles, derived from cancer cells, macrophages, erythrocytes, or platelets, leverage the homotypic targeting properties of the original cells, allowing for immune evasion and more natural interactions with immune cells [32,33]. This design enables these systems to home to tumor sites more effectively than traditional synthetic nanoparticles, providing a potential approach for tumor-targeted therapies [31,34].

Similarly, exosomes, which are natural nanocarriers used by cells for intercellular communication, offer an advantage due to their endogenous cargo-loading and targeting capabilities [35]. Their inherent ability to deliver gene therapy and immunotherapeutic agents makes them a highly effective vehicle for combined therapies, particularly in cancer immunotherapy [36,37,38]. Additionally, bacterial outer membrane vesicles (OMVs) are nano-sized vesicles naturally secreted by Gram-negative bacteria, which have been engineered to enhance antigen presentation and immune activation, particularly in the context of vaccines and cancer immunotherapy [39,40,41]. When hybridized with polymers, OMVs can be optimized to improve the delivery of therapeutic agents, demonstrating their potential for enhanced therapeutic efficacy [42]. These innovative systems are paving the way for novel treatment modalities that harness natural properties for more precise and effective therapeutic delivery.

Generally, each class of nanoparticle offers distinct benefits. Lipid-based nanoparticles excel in nucleic acid delivery, polymers provide mechanical stability and controlled release, inorganic nanoparticles enable imaging and photothermal or catalytic functions, and biomimetic nanoparticles achieve natural targeting and immune modulation. However, each class also carries limitations that restrict performance when used alone, which has driven rapid growth in hybrid systems that combine lipid fluidity, polymer strength, inorganic functionality, and biological mimicry into a single delivery platform.

## 3. Design and Properties of Hybrid Nanocarrier Systems

**Figure 1:**
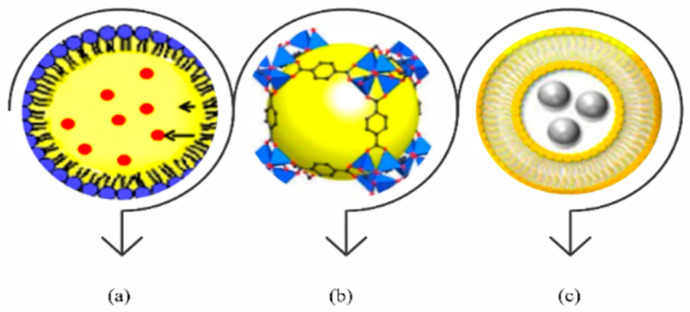
The architectures of hybrid nanocarriers: Flowchart of the main classes of hybrid nanocarriers: (**a**) lipid–polymer hybrid, (**b**) metal–organic frameworks, and (**c**) liposome–inorganic hybrid.

Robert Langer, a pioneer in drug delivery, describes hybrid nanocarriers as systems that unite lipids, polymers, and inorganic compounds. The goal is to improve stability, achieve targeted delivery, and control the release of therapeutic agents. This is especially valuable in cancer treatment. In simple terms, hybrid nanocarriers are made by combining two or more materials. Each material adds its own advantage. Together, they create a delivery system that is more efficient and precise. Several nanoparticle systems are already approved for cancer therapy [7]. These include liposomes, albumin-based carriers, and polymeric micelles. They are effective because they cross biological barriers, deliver drugs with accuracy, and release them in a controlled way. This helps maintain drug levels in the body over time [43]. Building on these successes, hybrid nanocarriers are now gaining strong attention. They are seen as a powerful option for delivering gene therapy and immunotherapy together. Both therapies are at the center of current research because they can improve outcomes and reduce side effects [44]. The logic of combining them is clear. Gene therapy targets cancer at the genetic level. It can silence or correct genes that drive tumor growth or resistance. Immunotherapy strengthens the immune system. It activates T cells and blocks pathways that suppress immune responses [45]. When given at the same time through one system, they work in harmony. The combination attacks cancer from different directions, making treatment more effective. For instance, siRNA can silence immunosuppressive genes. Along with immune checkpoint inhibitors, this can convert a “cold” tumor into a “hot” one. Such tumors are far more vulnerable to immune attack [5,46]. The challenge lies in controlling when and where drugs are released. Stability and safety in the body also remain critical. This is why advanced hybrid nanocarrier systems are essential. At present, several hybrid nanocarrier platforms have shown encouraging results in both lab studies and clinical trials. For instance, metal–organic frameworks (MOFs), with their tunable structures, have shown promise in preclinical studies for targeted cancer therapy, as demonstrated by Liang et al. (2021) [47]. Additionally, lipid–polymer hybrid nanoparticles (LPHNPs) have demonstrated high drug-loading efficiency and prolonged circulation time in preclinical studies, as shown by Tahir et al. (2019) [48]. These platforms fall into distinct categories, including LPHNPs, MOFs, and liposome–inorganic hybrids, as illustrated in Figure 1.

However, the vast majority of hybrid platforms reported to date remain at the preclinical stage, with only a small subset progressing into early-phase clinical testing, particularly in the context of oncology.

### 3.1. Lipid–Polymer Hybrid Nanoparticles (LPHNPs)

Lipid–polymer hybrid nanoparticles (LPHNPs) are an advanced type of nanocarrier. They consist of three main parts: First, a polymeric core that holds both hydrophobic and hydrophilic drugs. This core allows efficient loading and sustained release. Second, a lipid shell that surrounds the core. This shell improves biocompatibility, enhances stability, and reduces the incidence of premature drug release. Third, an outer layer of lipid–polyethylene glycol (PEG). This layer provides steric stabilization, extends circulation time, and helps the particles avoid immune clearance [49]. Because of this design, LPHNPs are highly versatile. They are useful in drug delivery, gene therapy, and imaging. Preclinical studies highlight their potential [48].

In contrast to traditional drugs, which are often clearly categorized as either hydrophobic or hydrophilic, gene therapy and immunotherapy rely on biologically active agents with a wide range of physical properties. These agents may be water-soluble, fat-soluble, or somewhere in between, depending on their structure and function. For instance, gene therapy typically uses nucleic acids such as DNA, RNA, siRNA, or mRNA—all of which are inherently hydrophilic [50]. This is due to the presence of negatively charged phosphate groups in their backbones, which interact easily with water molecules. In contrast, immunotherapy involves a broader set of molecules. Cytokines, interferons, monoclonal antibodies, and peptides are also generally hydrophilic because they are made of proteins. However, some small-molecule immunostimulants like toll-like receptor (TLR) agonists and lipid-based adjuvants are hydrophobic and do not mix well with water [51].

Because of this diversity in solubility and structure, delivering these therapies effectively poses significant challenges. To address this, researchers have developed lipid–polymer hybrid nanoparticles (LPHNPs), a multifunctional delivery platform designed to carry both hydrophilic and hydrophobic agents. These systems are especially valuable in overcoming solubility issues and biological barriers that often hinder the effectiveness of advanced therapies. In clinical development, lipid-based nanocarriers are among the most commonly used delivery systems. Typically, hydrophilic drugs are encapsulated in the central aqueous core, while hydrophobic drugs are embedded within the surrounding lipid layers [52]. One notable example is the use of hyaluronic acid-modified LPHNPs to simultaneously deliver Irinotecan, a chemotherapy drug, and plasmid DNA in colorectal cancer models. This co-delivery system achieved over 90% gene-loading efficiency and significantly enhanced the treatment’s ability to kill cancer cells [53]. Hyaluronic acid-modified nanocarriers enhance tumor targeting by exploiting overexpressed CD44 receptors on cancer cells, improving cellular uptake and increasing therapeutic efficacy. Hyaluronic acid (HA) is the principal ligand of CD44. CD44, an essential cell adhesion receptor expressed on cancer stem cells, features a specialized extracellular N-terminal domain responsible for HA binding within the extracellular matrix. Engagement of CD44 with HA triggers intracellular signaling pathways that drive cancer cell proliferation, invasion, and metastasis [54,55,56].

Currently, several clinical trials are evaluating the use of these hybrid nanoparticles for the targeted delivery of siRNA, particularly in combination with immunotherapeutic agents. This represents a promising approach for more precise, effective, and less toxic cancer treatments.

### 3.2. Metal–Organic Frameworks (MOFs)

Metal–organic frameworks (MOFs) are a highly adaptable class of nanomaterials known for their porous structures, tunable surface properties, and exceptional capacity to carry therapeutic agents [57,58]. Because of their structural flexibility, researchers have developed a wide variety of MOFs, including zeolite-like imidazolate frameworks (ZIFs), polymorphic structures such as Materials of Institute Lavoisier (MIL), MOFs containing alkaline earth metal frameworks (AEMFs), and rare-earth metal MOFs (RE-MOFs) [59]. RE-MOFs are particularly valued for their high coordination numbers, luminescent properties, and relatively low toxicity, making them attractive platforms for biomedical applications [60]. In parallel, transition-metal frameworks such as HKUST-type MOFs (based on copper paddlewheel units developed at the Hong Kong University of Science and Technology) have become well-established because of their large surface area, high porosity, and ease of functionalization [61]. These frameworks can be further enhanced by integrating them with other materials, broadening their utility in targeted delivery and combination cancer therapies [57].

In recent years, metal–organic frameworks (MOFs) have shown potential in delivering both gene therapy and immunotherapeutic agents simultaneously. Their multi-functional structure allows them to support dual-action therapies, which can amplify the overall therapeutic effect when treating tumors [62]. One practical application involves functionalized MOFs designed to interfere with genes that promote tumor growth or allow cancer cells to evade immune detection. For example, ZIF-8-based MOFs have been successfully used to deliver siRNA targeting PD-L1, a key immune checkpoint protein. This strategy has led to stronger T cell-mediated immune responses, helping the immune system better recognize and attack cancer cells [63]. A summary of the applications of MOF hybrids in ongoing preclinical and clinical studies is presented in Table 1.

### 3.3. Liposome–Inorganic Hybrids

The combination of liposomes and inorganic materials such as gold nanoparticles, silica, or iron oxide has led to the creation of liposome–inorganic hybrid nanocarriers, which offer the biocompatibility of liposomes and the unique physicochemical properties of inorganic materials. These hybrid systems represent a cutting-edge class of nanocarriers that are specifically engineered to address several key challenges in cancer therapy, including targeted drug delivery, controlled release, and the ability to support multimodal treatment strategies [68].

**Figure 2:**
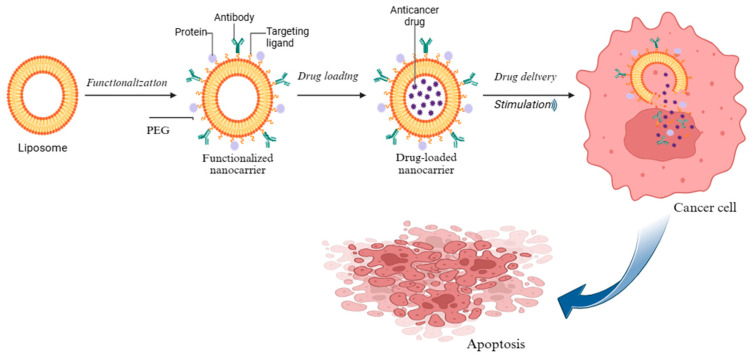
Schematic representation of the stepwise mechanism of drug delivery to cancer cells via liposome–inorganic hybrid nanocarriers.

Ordinarily, these nanocarriers feature an inorganic core encapsulated within a lipid bilayer, which may be functionalized to enhance targeting precision, increase structural stability, and improve overall therapeutic performance. To further improve their pharmacokinetics, polyethylene glycol (PEG) is often added to their surface. This PEGylation helps shield the particles from immune detection and reduces premature clearance from the bloodstream, thereby extending their circulation time [8]. As shown in Figure 2, the process of liposome–inorganic hybrid drug delivery begins with functionalization and drug loading, ultimately leading to the targeted delivery of therapeutic agents to cancer cells. This mechanism leads to the induction of apoptosis within the cancer cells.

One of the key benefits of liposome–inorganic hybrids is their ability to co-deliver multiple therapeutic agents with different mechanisms of action. For example, they can be used to transport photothermal agents like indocyanine green alongside traditional chemotherapeutics such as doxorubicin, enabling synergistic effects in gene-immunotherapy combinations [69].

Despite its benefits, PEGylation gives rise to the “PEG dilemma”, where the PEG coating reduces cellular uptake and intracellular delivery efficiency [70]. To overcome this, researchers have developed “de-PEGylation” techniques that use tumor-specific environmental triggers such as acidic pH or high redox conditions to detach the PEG layer. This is typically achieved through cleavable chemical bonds like hydrazone or disulfide bridges, allowing the nanocarrier to enhance cell interaction and effectively release its therapeutic contents at the tumor site [71].

### 3.4. Challenges and Translational Considerations

Hybrid nanocarriers hold significant promise in precision cancer therapy, particularly through their ability to co-deliver gene-editing agents and immunotherapeutic compounds. However, despite their potential, bringing these advanced systems into clinical use presents a number of interrelated challenges and practical considerations that must be addressed for successful translation from the lab to the clinic.

#### 3.4.1. Scalability and Batch Consistency

As earlier highlighted, hybrid nanocarriers integrate highly diverse and complex materials such as lipid–polymer composites or metal–organic frameworks. Thus, numerous challenges in maintaining consistent particle size, drug-loading efficiency, and ligand conjugation may likely be encountered. Even with strict control parameters, such as maintaining a low polydispersity index, manual synthesis methods often lead to batch-to-batch variability, particularly in lipid–polymer ratios. Microfluidic techniques have been explored to address this by enabling precise control over nanoparticle size (PDI < 0.1) and drug loading efficiency (>90%) [5]. Xu et al. [72] demonstrated that precise manipulation of flow rate ratio (FRR) and total flow rate (TFR) in a microfluidic flow-focusing device enabled controlled synthesis of polymer–lipid hybrid nanoparticles with tunable sizes. The resulting nanoparticles exhibited remarkable uniformity, maintaining a polydispersity index (PDI) below 0.1, which significantly outperformed conventional bulk mixing techniques. Notably, the method achieved high drug encapsulation efficiency, reaching up to 88% for doxorubicin, with a loading content of approximately 25%. In addition, the nanoparticles showed excellent colloidal stability and sustained drug release behavior, underscoring the potential of microfluidic platforms in precision nanomedicine design.

However, GMP-compliant manufacturing processes remain underdeveloped for clinical-scale production [73]. Furthermore, the multi-step fabrication processes involved in the manufacturing of hybrid nanocarriers, such as PEGylation and redox-sensitive crosslinking, result in significantly increased production costs and complicate scale-up.

#### 3.4.2. Biological and Physiological Barriers

Hybrid nanocarriers face significant challenges due to the inherent variability in human tumors and the complex tumor microenvironment (TME). Preclinical models often overestimate the enhanced permeability and retention (EPR) effect; however, human tumors display a wide range of vascular pore sizes (100–800 nm) and interstitial pressures (5–40 mmHg), which can limit the effective penetration of nanocarriers, particularly in dense or hypoxic regions. For instance, in pancreatic adenocarcinoma, only 12–18% of hyaluronidase-integrated hybrids penetrated hypoxic cores due to stromal density [74]. Murine models also poorly replicate human immune checkpoint expression (e.g., 68% PD-L1 homology), limiting translational relevance [75].

In addition, the TME presents its own set of challenges. For instance, acidic pH (6.5–6.9) and elevated glutathione (GSH: 2–10 mM) in the TME trigger premature payload release. Cationic polymer cores lose 40–60% of siRNA before cellular uptake in triple-negative breast cancer models, reducing gene silencing by 3.5 [76]. ABC transporters (e.g., P-glycoprotein) further diminish intracellular doxorubicin concentrations by 70% in multidrug-resistant cancers [77].

#### 3.4.3. Immune-Related Adverse Events (IRAEs)

Immune-related toxicity and biocompatibility remain significant challenges. The co-delivery of immunostimulatory agents such as TLR agonists and interleukins can trigger cytokine release syndrome in a notable number of cases. While hybrid nanocarriers reduce systemic toxicity, preclinical models report cytokine release syndrome (CRS) in 15–20% of cases following IL-12 or TLR agonist delivery [78]. PEGylation mitigates immunogenicity but may impair dendritic cell maturation, necessitating surface ligand optimization [79]. Furthermore, certain nanocarrier components, such as metal-based nanoparticles (e.g., TiO2 nanoparticles), iron oxide nanoparticles, and zinc oxide nanoparticles have been associated with oxidative stress, DNA damage, and mitochondrial dysfunction at higher doses, which may contribute to off-target toxicity in organs like the heart [80,81,82,83].

#### 3.4.4. Stability and Release Kinetics Challenges

Using hybrid nanocarriers to co-encapsulate both hydrophilic and hydrophobic payloads often results in mismatched release kinetics, and this can undermine the synergistic effects intended by the combined therapies. Notably, co-encapsulating hydrophilic CRISPR plasmids and hydrophobic immunotherapies (e.g., anti-CTLA-4) led to 25–40% payload leakage in serum within 6 h [84]. Mismatched release kinetics (e.g., gemcitabine t_1/2_= 2 h vs. miR-21 inhibitor t_1/2_ = 8 h) reduced synergy by 50% [85]. Additionally, stabilization techniques such as lyophilization may alter particle sizes, negatively impacting their therapeutic performance. The storage stability of sensitive components like siRNA is further compromised over time, as lipid peroxidation can degrade integrity, affecting the overall efficacy of the treatment. For instance, lyophilized lipid–polymer hybrids retained only 60–70% siRNA integrity after 6 months at 4 °C due to lipid peroxidation [86]. Trehalose stabilization increased particle size, compromising tumor penetration.

#### 3.4.5. Future Directions to Mitigate Challenges in Hybrid Nanocarriers

Future directions for hybrid nanocarriers involve several innovative strategies that can overcome current limitations and advance clinical translation. Integrating tumor microenvironment (TME) profiling has become a focus to many researchers, particularly through assessing PD-L1 expression and hypoxia markers in nanocarrier design which could enable them to develop patient-specific regimens, thereby enhancing treatment precision [87]. Additionally, incorporating multimodal imaging by combining MRI/CT-visible nanoparticles with therapeutic payloads offers the promise of real-time monitoring of tumor targeting and drug release, which could significantly improve delivery efficacy [88]. Finally, next-generation immune modulators are on the horizon, with approaches like co-delivery of STING agonists with CAR-T cells via biodegradable polymers may boost solid tumor infiltration and overall immunotherapeutic effectiveness [89].

In summary, each major hybrid platform discussed in this section offers a distinct balance of benefits and limitations. Lipid–polymer hybrids typically provide favorable pharmacokinetics and biocompatibility, with tunable cores for nucleic acids or small molecules and lipid shells that prolong circulation; however, their multi-component nature can complicate large-scale manufacturing and quality control [5,7,75]. MOF-based hybrids offer exceptionally high loading capacities and inherent catalytic or imaging functions, yet they raise particular concerns regarding long-term metal accumulation, biodegradation, and batch-to-batch reproducibility under physiological conditions [57,58,63,64]. Liposome–inorganic hybrids, including gold- and silica-based constructs, combine the biological familiarity of liposomes with photothermal or contrast-enhancing properties of inorganic cores, but they must be carefully engineered to avoid RES sequestration, chronic tissue deposition, and cost escalation associated with complex functionalization [8,68,69,70]. Recognizing these trade-offs is essential when selecting platforms for gene–immunotherapy co-delivery, especially in indications where long-term safety and cost-effectiveness are critical.

## 4. Biological Barriers and How Hybrid Nanocarriers Overcome Them

Nanoparticle-based delivery systems encounter multiple biological barriers before achieving therapeutic activity. Following systemic administration, nanoparticles are rapidly coated by serum proteins—including opsonins, complement factors, and immunoglobulins—which label them for clearance by the mononuclear phagocyte system (MPS), particularly liver Kupffer cells and splenic macrophages. The formation of this protein corona significantly shortens circulation half-life and reduces tumor bioavailability [90]. Surface engineering strategies such as PEGylation or biomimetic membrane cloaking can mitigate this effect. Hybrid nanocarriers coated with cell membranes or PEG layers exhibit reduced opsonin and complement binding, thereby prolonging systemic circulation and improving immune evasion [91].

A second major barrier arises after cellular uptake through endocytosis. Without efficient escape from endosomal compartments, nucleic acid therapeutics—including siRNA, mRNA, plasmids, and RNP complexes—remain trapped and are ultimately degraded.

To address this challenge, hybrid platforms increasingly incorporate pH-responsive or ionizable lipids, as well as proton-sponge polymers, that buffer endosomal acidification. This buffering effect drives proton and water influx, inducing osmotic swelling and subsequent endosomal membrane disruption, which enables cytosolic release of the therapeutic cargo [92].

A third set of challenges relates to pharmacokinetics and biodistribution, which are highly dependent on nanoparticle size, surface charge, mechanical stiffness, and the composition of the protein corona. Hybrid nanocarriers provide modular control over these parameters: a stealth or biomimetic outer layer—typically neutral or mildly anionic and well-hydrated—reduces rapid clearance, while the inner core is engineered to optimize cargo loading and controlled release. This tunable architecture enhances tumor accumulation through prolonged circulation and improved extravasation, ultimately increasing the therapeutic index relative to non-hybrid systems [93].

Collectively, these advantages—including enhanced immune evasion, efficient cytosolic delivery, and improved pharmacokinetics—underline the promise of hybrid nanocarriers for the co-delivery of gene therapeutics and immunomodulatory agents. Their modular and multifunctional properties position them as a powerful platform for next-generation cancer nanomedicine.

## 5. Comparison Between Hybrid and Non-Hybrid Nanocarrier Systems

Synthetic nanoparticles—including both conventional systems (e.g., liposomes and polymeric nanoparticles) and hybrid formats (such as lipid–polymer hybrids, inorganic–organic hybrids, and membrane-cloaked nanocores)—provide a versatile and complementary delivery strategy to viral vectors [94].

Viral vectors (including adeno-associated virus, lentivirus, and adenovirus) exhibit high transduction efficiency owing to evolutionarily optimized mechanisms for cellular entry and intracellular trafficking. However, their clinical utility is constrained by several well-documented limitations, such as immunogenicity, the generation of neutralizing antibodies that hinder repeat dosing, restricted cargo capacity, and the potential for insertional mutagenesis in integrating vectors. These challenges limit their broader application in oncology [95,96]. In contrast, synthetic nanocarriers circumvent genomic integration and enable modular, multi-cargo configurations. Hybrid architectures, in particular, decouple surface and core functionalities, facilitating the co-loading of chemically distinct payloads—for example, packaging nucleic acids within polymeric or inorganic cores while incorporating small-molecule immunomodulators into lipid shells. These platforms can further incorporate biomimetic coatings or stimuli-responsive elements to enhance immune evasion and achieve controlled, on-demand release [97].

Notably, the clinical success of lipid nanoparticle (LNP) platforms in mRNA vaccines, along with the advancement of LNP-based CRISPR therapeutics, demonstrates that non-viral delivery systems can achieve meaningful clinical efficacy in humans. Nonetheless, improving delivery efficiency for solid tumors and developing scalable, GMP-compliant manufacturing processes remain significant challenges that must be addressed to fully realize their translational potential [98].

## 6. Translational Outlook: From Bench to Bedside

Although most hybrid nanocarriers remain in preclinical development, several nanoparticle platforms—such as lipid nanoparticles (LNPs), metal–organic frameworks (MOFs), and inorganic–organic hybrid systems—have advanced from academic concepts to real-world clinical application. Their success demonstrates that nanoparticle-mediated immunotherapy and nucleic acid delivery can be safely and effectively translated to humans.

A major milestone was achieved with the approval of Patisiran (ONPATTRO^®^) in 2018, the first FDA-approved liver-targeted siRNA therapeutic to treat hereditary transthyretin (TTR) amyloidosis. Patisiran employs an LNP formulation (MC3/DSPC/cholesterol/PEG-lipid) that enables systemic delivery of siRNA to hepatocytes with acceptable safety and efficacy [99,100]. The global deployment of mRNA LNP vaccines during the COVID-19 pandemic further validated the scalability, manufacturability, and clinical safety of LNP-based technologies. This success accelerated the development of next-generation RNA therapeutics, including mRNA for protein replacement, immunomodulation, and cancer vaccination [101].

Beyond vaccines, LNP systems are increasingly used for therapeutic mRNA and gene-editing applications. New generations of ionizable lipid LNPs have been designed to enhance the safety and efficiency of nucleic acid delivery, including siRNA, mRNA, and CRISPR/Cas components [102].

One example under clinical investigation is BMS-986263—an LNP carrying siRNA targeting a fibrosis-related gene for the treatment of advanced liver fibrosis and cirrhosis [103].

Metal–Organic Frameworks (MOFs) are also gaining attention for their high surface area, tunable porosity, and substantial drug-loading capacity. For instance, a MOF-based delivery system for CRISPR/Cas9 gene editing is under investigation for improving gene-editing precision in cancer cells while minimizing off-target effects [65].

Despite these advances, several barriers remain. LNPs demonstrate efficient delivery to the liver and other accessible tissues, yet their performance in solid tumors and less-permissive organs remains modest. This limitation is driven by restricted biodistribution, poor tissue penetration, rapid MPS clearance, and challenges in producing consistent formulations at scale [104].

Hybrid nanocarriers—such as membrane-coated, ligand-targeted, or multi-component systems—may offer enhanced targeting and multifunctionality. However, these added layers of complexity can complicate large-scale manufacturing, regulatory approval, and reproducibility [105].

A central challenge is the intrinsic biodistribution restriction of LNPs. Many LNPs preferentially accumulate in hepatocytes due to interactions with serum apolipoproteins and natural clearance pathways. Recent studies have begun to elucidate the mechanisms underlying this organ tropism [90]. Efficient delivery to tumors, immune-privileged sites, and extrahepatic tissues remains limited—and may be further complicated by hybrid designs, which can alter biodistribution unpredictably [106].

Immunogenicity, reactogenicity, and innate immune activation also pose translational hurdles, especially with repeated dosing. Although LNPs enabled rapid deployment of mRNA vaccines, studies have shown that ionizable lipids and other LNP components can activate innate immune pathways—such as Toll-like receptors—leading to inflammation, cytokine release, and reduced tolerability [107]. Repeated or chronic therapeutic dosing amplifies these concerns, since these immune responses may undermine safety and therapeutic efficacy, in contrast to the generally well-tolerated, one-time vaccination setting. Furthermore, additional features in hybrid or targeted systems (e.g., ligands, membrane coatings) may modify immune recognition and increase immunogenic risk [108].

Even when cellular uptake is successful, endosomal escape and efficient cytosolic (or nuclear) release of the therapeutic cargo remain major bottlenecks—a challenge consistently highlighted across recent studies [92]. A systematic review of mRNA LNP intracellular trafficking highlighted that inefficient endo/lysosomal escape is a predominant obstacle for therapeutic applications requiring high protein expression or genome editing [109]. Complex hybrid carriers may further exacerbate this challenge, as additional lipid or polymer layers, surface coatings, or targeting moieties can interfere with membrane fusion, destabilization, and release kinetics [106].

Finally, long-term safety, stability, immune clearance with repeated administration, and unpredictable alterations in pharmacokinetics remain insufficiently explored.

While LNPs are generally well tolerated for single-use applications such as vaccination, repeated administration for chronic diseases, gene therapies, or immunotherapies introduces risks of cumulative toxicity, immune sensitization, and organ accumulation [110]. Hybrid carriers containing non-degradable components or multi-layered architectures may intensify these concerns, highlighting the necessity for rigorous long-term toxicology studies prior to clinical deployment [111,112].

## 7. Gene Therapy in Cancer Treatment

Gene therapy focuses on delivering genetic material into cells to correct genetic mutations or regulate biological pathways [113]. Key techniques include CRISPR/Cas9-mediated gene editing to precisely modify DNA, siRNA silencing to inhibit the expression of oncogenes, and the delivery of pro-apoptotic genes to trigger programmed cell death in cancer cells [114]. Additionally, gene therapy for cancer engages the immune system by reprogramming its components to overcome tumor-induced suppression and enhance antitumor responses. However, its success depends on addressing challenges posed by the immunosuppressive tumor microenvironment (TME) while managing potential side effects like Cytokine Release Syndrome (CRS) or immune exhaustion [115].

The treatment modality of gene therapy involves the use of nucleic acids to artificially modify the genetic expression of cells. In relation to cancer, considerable advances in understanding of the cellular genome have facilitated identification of the genes responsible for tumorigenesis and cancer’s resistance to certain therapies. With this genetic information, exogenous nucleic acid sequences can be designed and introduced into cells to modulate specific pathways to achieve antitumor effects. However, while gene therapy has the potential to revolutionize cancer treatment, the delivery of naked nucleic acids in vivo is hindered by several obstacles, including enzymatic degradation, poor tissue uptake, and a short blood half-life [116]. To address these challenges effectively, gaining a more thorough understanding of the precise structure of proposed vector complexes and their behavior under environmental stress would significantly aid in the rational development of such systems.

In addition to nanocarrier-based platforms, ligand-directed microRNA conjugate systems provide instructive examples of how small-molecule targeting can achieve highly specific gene delivery without a full nanoparticle scaffold. Recent work on a fully chemically modified miR-34a conjugated to a folate ligand (FM-FolamiR-34a) demonstrated selective accumulation in folate receptor–positive tumors, robust suppression of oncogenic pathways, and durable tumor regression in vivo [117]. Likewise, a PSMA-targeted, chemically stabilized miR-34a conjugate that combines a prostate-specific membrane antigen ligand with an endosomal escape moiety has achieved receptor-specific uptake and potent antitumor activity in prostate cancer models [118]. These minimalistic, receptor-guided constructs share key conceptual features with hybrid nanocarriers, including the decoupling of targeting, payload stabilization, and intracellular trafficking, and they illustrate how small-molecule ligands can be exploited to focus gene therapy on defined tumor subpopulations or immune niches, complementing more complex hybrid co-delivery systems.

One major challenge in the clinical application of hybrid nanocarriers, particularly those used for gene delivery, is the potential for unintended immune reactions or off-target effects. While these systems are designed to introduce genetic material into diseased cells, they can sometimes also affect healthy tissues, which raises concerns about safety [119]. In some cases, the introduced gene may integrate into the wrong location within the host’s DNA, leading to potentially harmful mutations.

This risk was highlighted in early clinical trials for X-linked severe combined immunodeficiency (X-SCID), where gene therapy using viral vectors led to serious complications. Specifically, two out of ten patients developed T cell leukemia due to unintended gene insertion events [120]. Another well-known example is the case of Jesse Gelsinger, an 18-year-old participant in a clinical trial aimed at treating ornithine transcarbamylase (OTC) deficiency. He was administered a recombinant adenoviral vector directly into his liver, but suffered a fatal immune response that brought attention to the significant risks associated with early gene therapy approaches. Tragically, Jesse died four days after the treatment [121]. The adenoviral vector provoked an unexpectedly severe immune response in his body, resulting in a cascade of multiple organ failures that ultimately caused his death [122]. A phase 3 trial targeting cerebral adrenoleukodystrophy (CALD) uses lentivirus-modified bone marrow stem cells to deliver a functional adrenoleukodystrophy protein (ALDP) gene, but safety concerns have arisen after one patient developed myelodysplastic syndrome (MDS), a potential precursor to leukemia, prompting the FDA to pause the trial as a precaution [121]. These examples underscore the urgent need for safe, more precise, and better-characterized delivery systems before widespread clinical adaptation.

Taken together, these examples underscore that the efficacy and safety of gene therapy are tightly constrained by delivery barriers, off-target risks, and immunotoxicity. Hybrid nanocarriers are particularly well-suited to address these issues because they can simultaneously protect nucleic acids from degradation, modulate biodistribution through surface engineering, and integrate stimuli-responsive release mechanisms that confine activity to the tumor microenvironment. In the context of this review, these features position hybrid systems as a rational platform for coupling gene modulation with immunotherapeutic interventions, rather than treating gene therapy as an isolated modality.

## 8. Immunotherapy in Cancer Treatment

Immunotherapy harnesses the body’s immune system to combat cancer through various strategies, including immune checkpoint inhibitors such as anti-PD-1 or anti-PD-L1 antibodies, which enhance the immune response against cancer cells [123]. CAR-T cell therapies involve engineering a patient’s T cells to recognize and destroy cancer cells, d and cytokine-based treatments, which boost the immune system by activating and regulating immune cells to target tumors effectively. Chimeric Antigen Receptor (CAR)-T and CAR-Natural Killer (CAR-NK) therapies are both cutting-edge immunotherapies designed to combat cancer by genetically modifying immune cells to target tumor-specific antigens [124]. While they share some similarities, they differ significantly in their mechanisms, safety profiles, and clinical applications [121]. The key differences between CAR-T and CAR-NK are summarized in Table 2.

Chimeric Antigen Receptor (CAR)-T cell therapy has emerged as a promising approach for targeted cancer treatment, involving the genetic modification of a patient’s T cells. While clinical trials have shown impressive results, the widespread application of this therapy faces challenges due to the complexity of current engineering methods, which rely on viral vectors and require extensive in vitro production of tumor-specific T cells. To address these limitations, researchers have turned to nanotechnology for in vivo T cell modification. Smith et al. [135] demonstrated the effectiveness of DNA-carrying nanoparticles (NPs) in delivering leukemia-targeting CAR genes directly into T cell nuclei, resulting in long-term disease remission. Their approach involved coating biodegradable poly (β-amino ester)-based NPs with anti-CD3 fragments to enable selective T cell uptake through receptor-mediated endocytosis. The NPs were loaded with plasmids encoding the leukemia-specific 194-1BBz CAR. This polymer-based NP system offers potential advantages in terms of simplified manufacturing and cost-effectiveness, making it a promising candidate for widespread leukemia treatment [135]. Another potential strategy involves using messenger RNA (mRNA) to induce transient CAR expression in T cells. Billingsley et al. developed ionizable lipid nanoparticles (LNPs) capable of delivering CAR mRNA to T cells. This method achieved CAR expression levels comparable to electroporation but with significantly reduced cytotoxicity. The LNP-based CAR-T cells demonstrated potent anti-cancer activity, suggesting a potential alternative to current engineering techniques [136]. These nanotechnology-based approaches offer promising solutions to overcome the limitations of conventional CAR-T cell engineering methods, potentially paving the way for more accessible and cost-effective cancer immunotherapies.

However, many immunotherapies still face limitations in terms of tumor infiltration, systemic toxicity, and treatment resistance driven by the tumor microenvironment. Hybrid nanocarrier systems provide a complementary strategy by enabling localized delivery of immunomodulators, controlled activation of innate and adaptive immune pathways, and co-packaging of genetic payloads that can reprogram immune cells or the tumor stroma. By framing immunotherapy within the context of hybrid co-delivery platforms, this review emphasizes not only the mechanisms of immune activation but also how advanced carrier design can overcome key translational bottlenecks.

## 9. Immune Suppression and Tumor Microenvironment (TME)

Tumor microenvironment (TME) is a dynamic and complex ecosystem that surrounds tumor cells, comprising a mix of immune cells, signaling molecules, stromal components, and extracellular matrix elements [137]. Cancer cells interact with this environment to promote their own survival and growth, often by creating conditions that suppress immune activity [138]. They achieve this by releasing immunosuppressive cytokines like TGF-β and IL-10, which dampen immune responses; recruiting regulatory T cells (Tregs) and myeloid-derived suppressor cells (MDSCs) to inhibit immune surveillance; and expressing immune checkpoint molecules such as PD-L1, which directly inhibit T cell function [139].

Given its central role in tumor cells, the TME has become a critical target in modern cancer therapy. Gene therapy offers powerful tools to disrupt the immunosuppressive nature of the TME. For example, strategies using CRISPR/Cas9 gene editing to knock out immune checkpoints like PD-1 in T cells have shown considerable promise. These modifications aim to boost immune cell activity by removing inhibitory signals that tumors use to escape immune attack [140].

One notable study demonstrated the effectiveness of CRISPR/Cas9-mediated PD-1 disruption in CAR-T cells [141]. Their findings showed that editing out PD-1 significantly enhanced the function of CAR-T cells in vitro, making them more resistant to PD-L1-mediated inhibition. In a subcutaneous xenograft mouse model, the edited T cells also showed improved tumor-clearing ability. Interestingly, the study revealed that tumors expressing high levels of PD-L1 could impair the function of second-generation anti-CD19 CAR-T cells and reduce their therapeutic effectiveness in vivo.

These insights underscore the importance of combining immunotherapy with gene editing techniques to better counteract the suppressive forces within the TME. They also highlight the need for continued research into integrated strategies that can reprogram immune cells to remain active and effective, even in hostile tumor environments.

## 10. Co-Delivery of Gene Therapy and Immunotherapy

Combination therapy has become a central strategy in cancer treatment, offering multiple benefits over single-agent approaches [142]. These include improved treatment outcomes, reduced toxicity, the ability to overcome multidrug resistance, and enhanced effects such as promoting apoptosis, inhibiting tumor metastasis and angiogenesis, triggering ferroptosis, and boosting antitumor immune responses [143]. Effective combination therapy depends on selecting agents with distinct mechanisms of action to avoid drug antagonism, proven synergistic effects in clinical settings, and minimal harm to healthy tissues to ensure safety [144].

In this context, co-delivering gene therapy and immunotherapy agents has gained attention as a powerful approach to attack cancer on two fronts by directly targeting tumor cells and modulating the tumor immune microenvironment. Hybrid nanocarriers play a key role in this co-delivery strategy by offering three major advantages [145]:

1. Encapsulation of multiple therapeutic agents;
2. Controlled and sequential release to optimize therapeutic effects;
3. Targeted delivery to enhance precision and reduce off-target toxicity.

Gene therapy and immunotherapy complement each other in powerful ways. Gene therapy works by delivering nucleic acids to either suppress oncogenes or restore the function of tumor suppressor genes [146]. Immunotherapy, in contrast, enhances the body’s immune response to detect and eliminate cancer cells. These therapies reinforce each other: Gene therapy can support immunotherapy by silencing immune checkpoints like PD-L1 or promoting cytokine expression, which activates immune cells [147]. In return, immunotherapy makes the tumor microenvironment more welcoming, which improves the delivery and effectiveness of therapeutic genes carried by gene therapy [148].

By combining both strategies, hybrid nanocarriers present an innovative and adaptable solution for precision cancer treatment. They can reprogram the tumor microenvironment, encourage immune cells to infiltrate tumors, and deliver therapies in a more targeted and coordinated way. A notable example is a hybrid lipid–polymer nanoparticle designed to carry both CRISPR-Cas9 and PD-1 siRNA into T cells. This system effectively knocks out PD-1, making immune cells less susceptible to suppression by PD-L1 and boosting their ability to attack tumors [149]. This dual-action approach makes immune cells more resistant to suppression by PD-L1 while increasing their tumor-targeting capability.

Mechanistically, the therapeutic benefit of co-delivering gene therapy and immunotherapy depends on more than simply packaging two agents into the same carrier. First, effective synergy requires that both payloads reach the same target cell population and, in many cases, the same subcellular compartment. For example, gene-editing tools such as CRISPR-Cas9 or siRNA must access the cytoplasm or nucleus, whereas checkpoint-blocking antibodies or small-molecule agonists act primarily at the plasma membrane or within endosomal signaling complexes. Hybrid nanocarriers can be engineered with compartmentalized architectures such as polymeric cores for nucleic acids and lipid shells for hydrophobic immune modulators to promote coordinated intracellular trafficking and co-localization at relevant sites of action [144,145,150]. Second, the temporal pattern of release is critical. In some settings, simultaneous release is desirable to ensure that immune activation occurs only when oncogenic signaling has been attenuated, whereas in others a sequential schedule (e.g., early knockdown of immunosuppressive genes followed by delayed checkpoint blockade) maximizes response. Core–shell hybrids and stimuli-responsive linkers (pH-, redox-, or enzyme-cleavable) enable such staggered release profiles by exploiting differences in degradation kinetics between layers or linkages [144,150]. Third, the stoichiometric ratio between gene and immune payloads strongly influences outcome; excessive immunostimulation in the absence of sufficient gene silencing may increase toxicity without durable tumor control, while too little immunotherapy undermines the benefit of precise genetic modulation. Hybrid systems facilitate rational tuning of this ratio by decoupling loading capacities in different domains (e.g., core versus shell) and by controlling surface ligand densities that regulate cellular uptake [16,145]. Together, these design levers—co-localization, release timing, and stoichiometry—provide a mechanistic framework for optimizing hybrid co-delivery platforms for different tumor and tumor microenvironment contexts.

**Figure 3:**
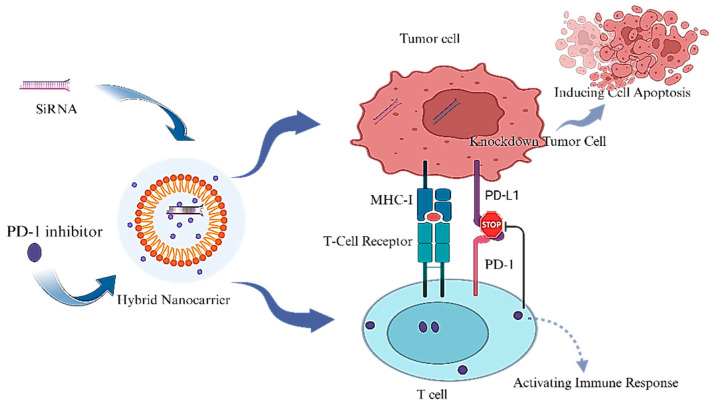
Schematic representation of a hybrid nanocarrier system designed for the concurrent and potentially sequential delivery of gene therapy and immunotherapy in tumor treatment. The system encapsulates PD-1 inhibitors and siRNA, targeting tumor cells by knocking down their expression, which activates T cells and triggers an immune response. This approach aligns with the complementary roles of gene therapy and immunotherapy. siRNA suppresses tumor cells, while PD-1 inhibitors enhance immune responses by blocking the interaction between PD-1 and PD-L1.

These systems are also highly effective at co-encapsulating both hydrophilic and hydrophobic agents, making them ideal for delivering gene-editing tools like siRNA or CRISPR-Cas9 alongside immune checkpoint inhibitors [150,151]. One standout innovation is the ZnPP@FQOS organosilica nanocarrier, which responds to acidic pH and high glutathione (GSH) levels in the tumor microenvironment. It releases quercetin, which remodels cancer-associated fibroblasts (CAFs) by downregulating Wnt16 and FAP-α, thereby reducing extracellular matrix (ECM) density and improving oxygenation. Simultaneously, zinc protoporphyrin (ZnPP) blocks heme oxygenase-1 (HO-1), boosting reactive oxygen species (ROS) generation during photodynamic therapy (PDT) [151]. This dual approach not only enhances PDT efficacy but also works in synergy with anti-PD-L1 immunotherapy, achieving remarkable 92.9% tumor suppression in pancreatic cancer models (Figure 3).

### Combined Therapeutic Impact of Immunotherapy and Gene Therapy

Immunotherapy has emerged as a leading breakthrough in cancer treatment and has been the focus of intensive research in recent years. Several types of cell-based immunotherapies have been developed, including Chimeric Antigen Receptor (CAR) T cells, T cell receptor-transduced T cells (TCR-T), tumor-infiltrating lymphocytes (TILs), and Natural Killer (NK) cells. Of these, CAR-T cell therapy has made the greatest clinical impact, with six treatments currently approved by the U.S. Food and Drug Administration (FDA) [152,153,154].

At the same time, immune checkpoint inhibitors (ICIs) have changed the way cancer is treated and are now a standard part of care for many types of tumors. Yet, despite these exciting advances, the success of immunotherapy can often be held back by the tumor’s own defenses [155]. The tumor microenvironment (TME) creates tough conditions such as low pH, lack of oxygen (hypoxia), and the presence of cells that suppress immune activity, which weaken the body’s ability to fight cancer [156].

This is where gene therapy holds promise. It can directly activate immune cells at the tumor site, boost the effectiveness of cancer vaccines, or restore immune functions that the TME has dampened [157]. One emerging strategy involves combining gene therapy with oncolytic virus therapy. These viruses are engineered to selectively infect and destroy tumor cells while delivering therapeutic genes that help break down immunosuppressive barriers and stimulate strong antitumor immune responses [158]. This combination offers a promising approach to overcoming the limitations of immunotherapy and improving clinical outcomes for patients with difficult-to-treat cancers.

In addition, nucleic acid-based vaccines, including mRNA vaccines, have become valuable and safe tools in cancer treatment because of their lasting effects. Using gene therapy to reshape the tumor microenvironment opens possibilities for making immunotherapy more effective and advancing cancer care. Table 3 below highlights hybrid nanocarriers that have been reported for gene therapy and immunotherapy delivery, showcasing their key components, therapeutic payloads, and reported therapeutic outcomes.

## 11. Targeting the Tumor Microenvironment with Advanced Hybrid Nanocarriers

One of the key strengths of hybrid nanocarriers lies in their ability to take advantage of the tumor microenvironment’s unique features, like its acidic conditions (low pH), low oxygen levels (hypoxia), and elevated enzymatic activity. This enables controlled and site-specific drug release, which minimizes off-target effects and systemic toxicity. Hybrid nanocarriers play a critical role in exploiting pH variations in the tumor microenvironment. Usually, tumors often exhibit an acidic extracellular pH (around 6.5–7.0) compared to normal tissues (pH 7.4) due to anaerobic glycolysis (the Warburg effect), and this poses a serious challenge that hinders anti-cancer drugs from exerting their function efficiently. Interestingly, with advancements in nanotechnology, hybrid nanocarriers can be engineered with pH-sensitive materials (e.g., acid-labile bonds or pH-responsive polymers) to trigger drug release in the acidic tumor microenvironment, ensuring site-specific delivery while sparing healthy tissues. Wang et al. 2022 [165] demonstrated how polymeric–lipid hybrid nanoparticles successfully delivered siRNA to target PD-L1 and dendritic cell activators. In their work, they showed that polymeric cores degrade in acidic environments to release both agents to suppress immune checkpoints and boost dendritic cell activation. This yields significant immune activation and tumor suppression in murine colorectal cancer models.

### 11.1. Heterogeneity of the Tumor Microenvironment and Implications for Drug Delivery

**Figure 4:**
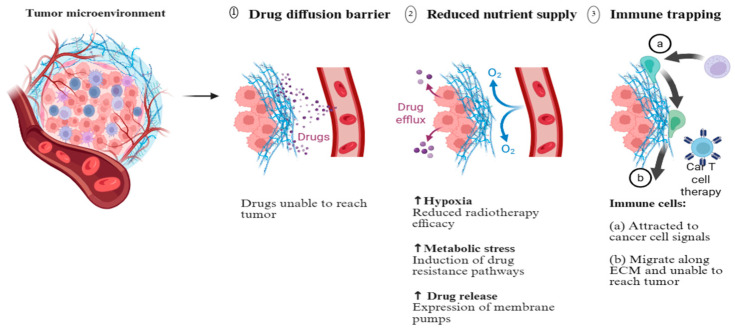
Key features of the tumor microenvironment affecting drug delivery.

The TME is characterized by abnormal vascularization, hypoxia, acidic pH, and dense fibrotic networks orchestrated by cancer-associated fibroblasts (CAFs) [151]. These features create a physically and biochemically immunosuppressive milieu that limits the penetration and efficacy of conventional therapeutics. For instance, the irregular vasculature of tumors enables passive accumulation of nanocarriers via the enhanced permeability and retention (EPR) effect, but heterogeneous pore sizes (100–800 nm) and elevated interstitial fluid pressure often restrict uniform distribution [5]. This complexity is illustrated in Figure 4, which depicts the various barriers to drug diffusion in the TME. Hybrid nanocarriers address these challenges through size-tunable architectures (typically 50–200 nm) and surface modifications that enhance vascular extravasation and deep tissue penetration [5]. For example, PEGylated lipid–polymer hybrids exhibit prolonged circulation times (>3 h) and preferential accumulation in pancreatic tumors via EPR, as demonstrated in Panc-1 xenograft models [5].

### 11.2. Active Targeting of TME-Specific Biomarkers

Beyond passive accumulation, hybrid nanocarriers employ ligand-mediated active targeting to home in on overexpressed receptors in the TME. Transferrin, folic acid, and hyaluronic acid are widely used ligands that bind to receptors upregulated on tumor cells and stromal components [16]. In glioblastoma models, transferrin-conjugated lipid–polymer nanoparticles co-delivering doxorubicin and erlotinib achieved a 4.7-fold higher accumulation in brain endothelial cells compared to non-targeted systems [5]. Similarly, folic acid-decorated hybrids enhanced daunorubicin and cytarabine delivery to CD44-overexpressing acute myeloid leukemia cells, reducing off-target toxicity [5]. These strategies exploit the TME’s metabolic demands—such as elevated iron uptake in proliferating cells—to improve therapeutic specificity.

### 11.3. Reprogramming Immune Cell Dynamics

The immunosuppressive nature of the tumor microenvironment (TME) remains one of the major obstacles to achieving long-term success with cancer therapies. This environment is often characterized by the presence of M2-polarized tumor-associated macrophages (TAMs), regulatory T cells (Tregs), and a lack of cytotoxic T lymphocyte (CTL) infiltration, all of which contribute to poor immune responses and therapeutic resistance [61]. Hybrid nanocarriers offer a powerful tool to reprogram this hostile environment. When loaded with toll-like receptor (TLR) agonists like CpG oligonucleotides or cytokine modulators such as interleukin-12 (IL-12), these systems can reprogram TAMs into pro-inflammatory M1 macrophages and promote the maturation of dendritic cells (DCs)—key steps in activating an effective antitumor immune response [166].

A compelling example is the use of polymer–lipid manganese dioxide nanoparticles (PLMD-NPs), which are engineered to scavenge excess hydrogen peroxide in the TME. By alleviating hypoxia, these nanoparticles help reverse PD-L1-mediated T cell exhaustion and improve the therapeutic effect of doxorubicin in breast cancer models [167].

In parallel, gene-silencing approaches using siRNA-loaded hybrid nanocarriers have shown great potential. Delivering siRNA targeting STAT3 or NF-κB via cationic polymer cores helps inhibit key immunosuppressive signaling pathways within the tumor. This not only enhances immune activation but also restores CTL-driven tumor cell killing, improving overall treatment efficacy [150].

### 11.4. Overcoming Extracellular Matrix (ECM) Barriers

The dense ECM in desmoplastic tumors like pancreatic and colorectal cancers physically impedes nanocarrier diffusion and fosters chemoresistance [151]. Hybrid systems incorporating collagenase or hyaluronidase degrade ECM components to enhance intratumoral permeability. In a KP fibroblast-rich tumor model, quercetin-loaded ZnPP@FQOS reduced collagen I and fibronectin deposition by 68%, significantly improving nanocarrier penetration and ROS diffusion [84]. Alternatively, CAF-targeted hybrids delivering TGF-β inhibitors disrupt fibroblast activation, mitigating ECM production and stromal stiffness [168].

### 11.5. Considerations of Tumor Microenvironment–Targeted Strategies

Hybrid nanocarriers appear highly promising in preclinical studies. However, their translation into clinical practice remains a challenging task. The main limitations include batch-to-batch variability during synthesis, concerns regarding long-term safety, and difficulties associated with large-scale production [169]. In addition, interpatient heterogeneity represents a substantial obstacle, as differences in the tumor microenvironment (TME) require individualized ligand selection and dosing strategies. The application of emerging technologies, such as microfluidic platforms and computational modeling, offers promising opportunities to address these challenges by enabling high-throughput manufacturing with improved control over drug loading ratios and release kinetics. For example, polymer–lipid hybrid nanocarriers containing redox-sensitive disulfide bonds selectively release their therapeutic payload within tumor cells characterized by elevated glutathione (GSH) levels, thereby minimizing off-target toxicity to healthy tissues [150].

The TME is highly complex and dynamic. Hybrid nanocarriers are well-suited for this because they can combine multiple functions. They can deliver drugs, edit genes, and modulate immunity within one system. Future research should focus on biomarker-based patient selection. It should also explore combinations that take advantage of sequential control over TME components. As we learn more about interactions between tumor, stroma, and immune cells, these technologies may help overcome resistance and achieve longer remissions.

At the same time, clinical and translational experience has revealed that TME-targeted strategies do not uniformly translate into improved outcomes. The magnitude of the EPR effect in human tumors is often lower and more heterogeneous than in preclinical models, leading to modest or variable nanocarrier accumulation despite encouraging animal data [5,16,167]. Similarly, interventions aimed at depleting stromal components or carcinoma-associated fibroblasts (CAFs) can have paradoxical effects. For example, genetic or pharmacologic depletion of CAFs in pancreatic cancer accelerated disease progression and reduced survival in some models, highlighting the protective as well as tumor-promoting roles of certain stromal populations [60,151]. These observations underscore the need for hybrid nanocarrier designs that modulate, rather than indiscriminately ablate, TME components, and for careful stratification of patients based on stromal and immune phenotypes.

### 11.6. Practical Framework for Selecting Hybrid Nanocarrier Platforms

The choice of hybrid nanocarrier platform should be guided by both the biological context of the tumor microenvironment (TME) and the therapeutic goals of the co-delivery strategy. Although hybrid systems offer powerful capabilities, they also introduce additional complexity in design, manufacturing, and regulatory evaluation [5,16]. It is therefore important to identify scenarios in which their advantages are likely to outweigh those of simpler formulations.

From a TME perspective, desmoplastic and ECM-rich tumors (e.g., pancreatic and certain colorectal carcinomas) are characterized by dense stroma, elevated interstitial pressure, and poor perfusion, all of which restrict nanoparticle penetration [151]. In these settings, hybrid platforms that integrate ECM-modulating functions such as organosilica hybrids co-delivering quercetin and photodynamic agents, or systems incorporating collagenase or hyaluronidase, are particularly attractive because they can simultaneously remodel the matrix and enhance intratumoral distribution of co-delivered gene and immunotherapy payloads [151,168]. By contrast, in more highly vascular, EPR-favorable tumors with relatively loose stroma, simpler lipid or polymeric nanocarriers may be adequate unless additional capabilities (e.g., built-in imaging or multi-stage release) are required [5,16,167].

For immune-excluded or strongly immunosuppressive TMEs, where cytotoxic lymphocytes fail to infiltrate deeply, hybrid platforms that combine immune modulation and gene regulation are often justified. Examples include systems co-delivering TLR agonists or STING agonists with siRNAs targeting STAT3, PD-L1, or other checkpoints, or hybrids that alleviate hypoxia via MnO_2_ or ROS-modulating components while simultaneously delivering chemotherapeutics or gene-editing tools [150,151,166,167]. In such contexts, the ability of hybrid systems to coordinate multiple mechanisms, such as ECM remodeling, hypoxia relief, checkpoint inhibition, and vaccine-like antigen presentation, can produce synergistic immune activation that is difficult to achieve with a single-modality carrier.

Therapeutic intent also plays a key role. When the goal is simple gene silencing or delivery of a single cytokine to a well-perfused tumor expressing a well-validated receptor, ligand-directed conjugates or conventional nanocarriers may offer sufficient selectivity with lower complexity and cost. In contrast, co-delivery scenarios that require (i) precise control of the ratio between gene and immunotherapy agents, (ii) sequential or compartmentalized release, or (iii) integration of diagnostic imaging and therapy (theranostics) strongly favor hybrid designs such as lipid–polymer hybrids, MOFs with catalytic or imaging cores, or liposome–inorganic hybrids that support photothermal or photodynamic functions [5,62,68,146,169].

Finally, patient-to-patient variability in TME features highlights the need for a flexible, modular design logic. One practical approach is to start from a “base” platform (e.g., lipid–polymer hybrids for nucleic acid and antibody co-delivery) and layer additional functions such as ECM-degrading enzymes, hypoxia-responsive linkers, or imaging agents only when dictated by tumor histology, stromal density, and immunophenotype [5,16,151]. In this way, the added complexity of hybrid nanocarriers is reserved for indications where it is most likely to improve clinical benefit, while simpler systems remain preferable when the biological and therapeutic requirements are less demanding.

## 12. Preclinical Applications in Gene and Immunotherapy Co-Delivery

Emerging preclinical research continues to highlight the potential of hybrid nanocarriers as delivery systems for combining gene therapy with immunotherapy. The examples summarized in this section are predominantly preclinical studies in murine or other animal models, and they should therefore be interpreted as proof-of-concept demonstrations rather than established clinical therapies. Potential and striking examples are in the pipeline, as briefly highlighted below.

### 12.1. CRISPR-Cas9 and Immune Checkpoint Inhibitors

Cationic lipid nanoparticles co-delivering CRISPR-Cas9 components and anti-PD-L1 antibodies have shown efficacy in melanoma and triple-negative breast cancer models. In B16F10 melanoma-bearing mice, this system achieved 80% tumor regression by simultaneously knocking out PD-1 in T cells and blocking PD-L1 on tumor cells [145]. The combination increased tumor-infiltrating CD8+ T cells by 3.2-fold compared to monotherapy [170].

### 12.2. siRNA and Cytokine Combinatorial Therapy

pH-sensitive polymeric nanocarriers co-loaded with STAT3 siRNA and interleukin-12 (IL-12) reversed immunosuppressive TMEs in the 4T1 mammary carcinoma model. STAT3 silencing reduced Treg infiltration by 67%, while IL-12 polarized macrophages to the M1 phenotype, enhancing dendritic cell activation [150]. This strategy reduced metastatic lung nodules by 89% and extended survival from 28 to 52 days.

### 12.3. Antitumor Nanovaccines Enhanced by siRNAs

Therapeutic cancer vaccines are designed to stimulate the immune system to recognize and eliminate cancer cells by activating immune cells such as CD8+ cytotoxic T cells and CD4+ helper T cells. Their effectiveness can be significantly improved with the use of nanoparticles (NPs), which enhance the delivery and presentation of cancer antigens to immune cells, making it easier for the immune system to recognize tumor-specific targets [171].

Nanoparticles achieve this by transporting cancer-associated peptides or genetic material like mRNA and DNA into immune cells. Once internalized, these genetic payloads are processed to produce antigenic peptides that trigger a strong and specific immune response [79]. Incorporating multiple tumor antigens into a single nanoparticle formulation also increases the vaccine’s precision and therapeutic reach.

A compelling example of this strategy comes from Huang et al. [172], who developed lipid dendrimer-based nanoparticles specifically targeting hepatocellular carcinoma (HCC). Their system co-delivered an HCC-specific antigen, PD-L1-targeting siRNA, and a gene encoding interleukin-2 (IL-2). This combination not only boosted T cell activation but also effectively inhibited tumor progression and metastasis in animal models.

## 13. Future Perspectives

Looking forward, the future of hybrid nanocarriers in precision oncology lies in their ability to evolve into multifunctional platforms that seamlessly integrate targeted delivery, stimuli-responsive drug release, real-time imaging, and computational design. This convergence of technologies is expected to overcome many of the current challenges in cancer treatment while significantly enhancing therapeutic precision, safety, and overall clinical outcomes.

### 13.1. Advanced Biomimetic and Targeted Delivery Platforms

Next-generation hybrid nanocarriers are expected to increasingly utilize cell-specific targeting mechanisms and biomimetic surface coatings to improve tumor localization and evade immune detection. One promising example involves organosilica-based nanocarriers engineered with PEGylated ligands, which promote selective uptake by tumor cells while minimizing interaction with immune cells such as macrophages. In a recent study, this design enabled the sequential delivery of doxorubicin to tumor cells and the immunostimulant resiquimod to immune components, resulting in an effective chemoimmunotherapy strategy. Remarkably, this approach achieved up to 70% tumor suppression in preclinical cancer models [173]. Sancho-Albero et al. [173] highlighted the therapeutic potential of precisely targeted and functionally layered nanocarrier systems. Similarly, DNA-based “nanokites” co-delivering p53 genes and doxorubicin achieved 80% tumor regression in multidrug-resistant cancers by exploiting ligand–receptor interactions [174].

In terms of ligand–receptor interactions, specific ligands can be designed to recognize overexpressed receptors on tumor cells, improving targeted drug delivery. For example, transferrin is used as a ligand that targets the transferrin receptor (TfR), which is commonly found on tumor cells due to their high iron demand for rapid growth [175]. Additionally, HER2-targeted nanocarriers, which use anti-HER2 antibodies or aptamers, specifically bind to the HER2 receptor that overexpressed on HER2-positive breast cancer cells, significantly improving chemotherapeutic agent delivery [176].

Future designs may integrate cancer stem cell membrane coatings or aptamer-guided systems (e.g., anti-HER2 DNA aptamers) to penetrate immunosuppressive niches and activate T cell infiltration.

### 13.2. Multimodal Stimuli-Responsive Systems

Next-generation hybrids will employ multi-stimuli activation for spatiotemporal control over gene and immunotherapy release. pH/enzyme dual-responsive chitosan–gold nanogels degraded in acidic tumor microenvironments (TME), releasing 87% of doxorubicin while enabling CT imaging-guided therapy [71]. Redox-sensitive Au-CGKRK nanoconjugates delivering PD-L1 and STAT3 siRNA enhanced survival by 70% in melanoma models through synchronized inhibition of immune checkpoints and JAK–STAT pathways [177]. Future iterations could combine photodynamic therapy (PDT) with immunomodulation–MnO_2_-embedded hybrids that scavenge ROS to amplify PDT efficacy while polarizing macrophages to pro-inflammatory M1 phenotypes [79].

### 13.3. Integrated Theranostic Platforms

In the evolving landscape of nanomedicine, theranostic hybrids stand at the forefront of innovation, integrating both therapeutic and diagnostic capabilities into a single powerful platform. These hybrid systems not only deliver targeted treatments but also provide real-time insights through diagnostic imaging, empowering clinicians to monitor and adjust therapies with unparalleled precision. Theranostic hybrids merging therapy and real-time imaging will dominate future pipelines. Gold nanoparticle–chitosan composites (CTPA@DOX) provided CT-guided tracking of drug accumulation while achieving >90% tumor growth inhibition [178]. Upcoming systems may integrate MRI-active gadolinium with near-infrared (NIR) dyes for precision photothermal therapy (PTT), as demonstrated by NIR-guided PTT reducing melanoma volume by 70% [179]. These platforms will enable adaptive dosing and real-time monitoring of immune activation.

### 13.4. AI-Driven Computational Design and Personalization

In this present era of nanotechnological advancement, artificial intelligence (AI) has revolutionized the design and optimization of hybrid nanocarriers, moving the process away from traditional trial-and-error methods to more efficient and predictive modeling [180]. As shown in Figure 4, the design process starts with the use of computational tools like AdaBoost https://scikit-learn.org/stable/modules/generated/sklearn.ensemble.AdaBoostClassifier.html (accessed on 19 December 2025), which employ machine learning algorithms to identify the most effective material combinations for targeted cancer therapies. These AI-driven algorithms simulate and predict the ideal particle size, surface charge, and release patterns, all in silico, ensuring that the resulting nanocarriers are pre-optimized for specific tumor microenvironments (TMEs) [181]. During the synthesis of these nanocarriers, Internet of Things (IoT)-based monitoring systems offer continuous feedback, allowing researchers to make real-time adjustments to nanoparticle features. Automated data acquisition platforms feed this feedback directly back into AI models, creating dynamic loops that shorten the experimental cycle, reduce variability, and enhance the accuracy of the final product. This iterative process is key to achieving the precision seen in AI-driven nanocarrier systems. Importantly, these same tools can be directly aligned with the manufacturing and regulatory challenges discussed earlier by using model-based control to keep critical quality attributes (such as size distribution, drug loading, and surface charge) within GMP-compliant specifications and by predicting how design changes may influence in vivo behavior and safety profiles.

**Figure 5:**
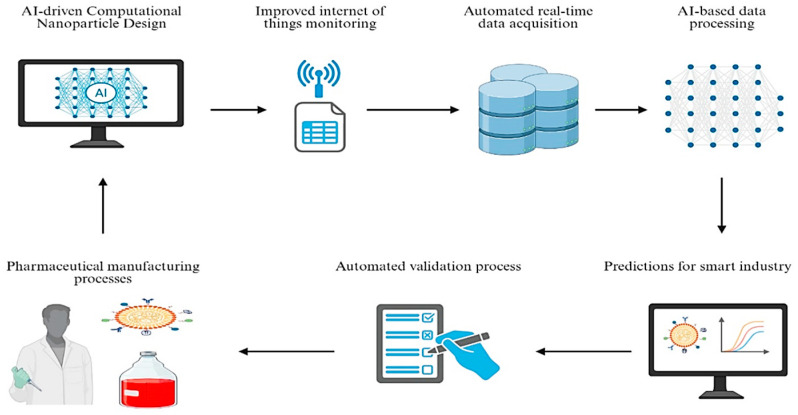
AI-driven computational design and personalization of hybrid nanocarrier-based therapies.

AI also plays a critical role in advancing the design process, as demonstrated in Figure 5, which represents how raw inputs such as patient-specific data or tumor biomarkers are interpreted to create customized formulations. These carriers can be tailored for specific applications, such as CRISPR-Cas9 and anti-PD-L1 hybrids for PD-L1-expressing tumors, or gelatin-based nanocarriers responsive to matrix metalloproteinases in fibrotic tissues [173]. This personalized approach ensures that each nanocarrier addresses the unique biological challenges of its target TME.

Beyond laboratory settings, AI is crucial in forecasting the behavior of these nanocarriers during the scale-up and manufacturing stages. By predicting both therapeutic performance and industrial feasibility, AI models help to bridge the gap between preclinical research and clinical translation [182]. Such in silico prediction and validation frameworks are likely to become increasingly important in regulatory submissions for complex hybrid nanocarriers, particularly those incorporating inorganic or MOF components, where long-term biodistribution and clearance are difficult to assess empirically.

As depicted in Figure 5, the final validation step ensures that the computationally designed nanocarriers remain consistent and effective under real-world conditions.

### 13.5. Scalable Manufacturing and Toxicity Mitigation

Addressing batch variability and toxicity remains critical. Microfluidic reactors enabled GMP-compliant synthesis of lipid–polymer hybrids with PDI < 0.1 [181]. CD47-mimetic peptide coatings reduced macrophage clearance by 38% without compromising targeting [177]. Future efforts will prioritize continuous-flow synthesis for metal–organic frameworks (MOFs) and lyophilization protocols to enhance storage stability. For hybrid systems containing inorganic or MOF-based cores, long-term safety and immunogenicity require special attention. The potential for chronic accumulation of heavy metals or non-biodegradable fragments, unforeseen immune activation, and organ-specific toxicity necessitates early integration of advanced toxicology studies including immunotoxicity, genotoxicity, and long-term biodistribution into the development pipeline [58,63,66]. Moreover, the added synthetic complexity of these platforms can significantly increase manufacturing costs relative to simpler liposomal or polymeric carriers, which has implications for scalability and patient access. Combining AI-guided optimization with health-economic and regulatory considerations from the outset may therefore be essential to ensure that clinically promising hybrid nanocarriers are also economically and logistically viable.

Of note, the future of hybrid nanocarriers lies in intelligent, patient-specific systems integrating biomimetic targeting, multi-stimuli responsiveness, and theranostics. By harmonizing computational design with advanced manufacturing, these platforms will transition from preclinical promise to clinical reality, offering curative outcomes in precision oncology. Collaborative efforts among material scientists, immunologists, and clinicians will be pivotal in overcoming scalability and regulatory challenges.

## 14. Conclusions

The integration of hybrid nanocarrier systems represents a transformative development in modern precision oncology, enabling the co-delivery of gene therapy and immunotherapy agents to address the persistent limitations of traditional cancer treatments. Hybrid carriers, whether lipid–polymer hybrids, metal–organic frameworks (MOFs), or liposome–inorganic combinations, are engineered to enhance targeting accuracy, improve therapeutic synergy, and reduce off-target toxicity. Notably, these platforms allow for the encapsulation of diverse therapeutic agents, both hydrophilic and hydrophobic, thus optimizing drug loading and controlled release profiles under physiological conditions.

Recent innovations such as smart stimuli-responsive mechanisms, biomimetic surface designs, and the application of artificial intelligence for precision optimization highlight the rapid progress in this field. Nanobiotix has validated the mechanism of action of its new class of radioenhancer, NBTXR3 (Hensify), which holds the potential to expand clinical applications in soft-tissue sarcoma. NBTXR3 is a hafnium oxide nanoparticle with smart stimuli-responsive mechanisms that enhance radiotherapy. When exposed to ionizing radiation, it produces reactive oxygen species, increasing tumor cell death while minimizing damage to nearby healthy tissue [183].

In preclinical and early clinical settings, hybrid nanocarriers have demonstrated the capability to overcome key delivery barriers, increase tumor selectivity, and induce more robust anti-cancer immune responses. Nevertheless, most gene–immunotherapy co-delivery systems described in this review have not yet advanced beyond preclinical evaluation, and only a limited number of hybrid nanocarriers are currently being tested in early-phase clinical trials. Ashton et al. [184] explored preclinical application of Accurins, a hybrid nanoparticle that encapsulates charged drugs via ion pairing. They demonstrated that when an Aurora B kinase was incorporated into the Accurins, it showed a significantly improved therapeutic index and better preclinical efficacy in rats and mice with human tumors, compared to the original molecule.

Despite these advances, several challenges must be addressed to fully realize clinical translation. A priority is the development of scalable, GMP-compliant manufacturing processes capable of producing nanocarriers with consistent size, composition, and therapeutic efficacy. Overcoming the biological complexities of the tumor microenvironment, such as heterogeneity in vascularization, stromal density, and immune checkpoint expression, remains essential for achieving predictable biodistribution and therapeutic benefit in patients.

Going forward, further engineering and process optimization are needed to enable reliable large-scale clinical production of hybrid nanocarriers. Automating synthesis and characterization protocols will be crucial for ensuring sterility, batch consistency, and cost-effectiveness in the production pipeline. Ultimately, these efforts are expected to accelerate the integration of hybrid nanocarrier technologies into routine clinical practice, supporting more personalized, targeted, and effective cancer therapies in the years to come.

## Figures and Tables

**Table 1:** Summary of applications of MOF hybrids.

Application Area	Payload Delivered by MOF Hybrids	Therapeutic Outcome	Reference(s)
Gene Therapy	siRNA (e.g., PD-L1, IDO1), CRISPR/Cas9	Reduced immune suppression, enhanced tumor regression	[64,65]
Immuno- therapy	Cancer vaccines, anti-PD-L1 antibodies	Enhanced APC activation, improved T cell-mediated immunity	[62,66]
Combined Gene- Immuno- therapy	siRNA + cancer vaccines	Synergistic effects on tumor growth inhibition	[67]
Multimodal Therapy	PDT + gene therapy; CDT + immunotherapy	Immunogenic cell death, enhanced antigen presentation	[47]

**Table 2:** An overview of differences between CAR-T and CAR-NK therapies.

Features	CAR-T	CAR-NK	References
Cell Type	T cells (adaptive immune system)	Natural Killer (NK) cells (innate immune system)	[125,126,127]
Mechanism of Action	CAR-T cells rely on CAR-specific recognition of tumor antigens for cytotoxicity	CAR-NK cells use both CAR-dependent and innate immune mechanisms to kill tumors	[128,129]
Cytokine Release Syndrome (CRS)	High risk of CRS due to excessive cytokine production during activation	Minimal or no CRS because NK cells produce fewer pro-inflammatory cytokines	[130,131]
Neurotoxicity	Associated with immune effector cell-associated neurotoxicity syndrome (ICANS)	Rare or absent neurotoxicity	[132]
Persistence in Body	Long persistence, which can lead to prolonged on-target off-tumor effects	Shorter lifespan, reducing risks of long-term toxicity but limiting durability	[133,134]
Efficacy Against Tumors	Highly effective in hematological malignancies but less so in solid tumors due to poor tumor infiltration and immunosuppressive microenvironments	Potential outcomes in both hematological and some solid tumors, with better tumor trafficking	[129]

**Table 3:** Examples of recently reported hybrid nanocarriers for the delivery of gene therapy and immunotherapy.

Hybrid NP System	Key Components	Cargo	Target or Indication	Mode of Action	Reported Therapeutic Outcome	Reference
T cell–macrophage hybrid membrane-coated ZIF-8	ZIF-8 core, T lymphocyte + macrophage hybrid membrane shell	siRNA against IRF1	M1 macrophages, autoimmune myocarditis model	pH-responsive endo-lysosomal escape, IRF1 knockdown, inhibition of macrophage pyroptosis	Reduced myocarditis progression in EAM mice without evident side effects	[159]
OMV–cancer cell hybrid membrane-coated PLGA NPs	PLGA core, hybrid bacterial outer membrane vesicle + cancer cell membrane	IR780 photosensitizer	Breast cancer bone metastasis, sonodynamic therapy	Tumor targeting and immune stimulation from OMVs, ROS generation under ultrasound via IR780	Inhibition of bone metastasis progression in mouse models	[160]
M1-polarized macrophage-derived cellular nanovesicle-coated LNPs (M1-C-LNPs)	Lipid nanoparticle (LNP) core, M1 macrophage-derived cellular nanovesicle (M1-NVs) shell	Bcl2-targeting siRNA, immune-modulating cytokines	Solid tumor, cancer immunotherapy and gene therapy	Bcl2 gene silencing and apoptosis induction in cancer cells, immune modulation via M1-NVs, tumor retention via adhesion molecules	Superior tumor growth inhibition, enhanced intratumoral immune response, granule-mediated tumor cell killing, effective gene-immunotherapy combination	[161]
Hybrid nanoparticle-based in situ vaccine (ISV)	Diselenide-bridged organosilica NP core, Mn^2+^-based metal–phenoc network shell	SN38 (chemodrug)	Dendritic cells in tumor microenvironment, cancer immunotherapy	Mn^2+^ and SN38 co-activate the STING pathway, diselenide + polyphenol scavenge ROS, protect DCs from oxidative damage	Activated DCs, enhanced T cell activation, induced systemic antitumor immunity, improved ISV efficacy	[162]
Platelet–glioma hybrid membrane-camouflaged nanoparticles	Hybrid membrane from platelets + glioma cells, polymeric NP core	Model anti-cancer payloads	Glioma targeting and immune evasion	Homotypic binding via glioma membrane proteins, immune escape via platelet markers	Enhanced tumor accumulation and antitumor efficacy in glioma models	[163]
Cancer cell–macrophage hybrid membrane-coated CuS NPs with anti-VEGFR	Hollow CuS core, hybrid CCM + macrophage membrane, anti-VEGFR antibody	Sorafenib	Hepatocellular carcinoma	Photothermal therapy plus chemotherapy plus anti-angiogenesis, immune evasion and homotypic targeting	Sustained tumor growth suppression and reduced metastasis in HCC models	[164]

## Data Availability

No new data were created or analyzed in this study. Data sharing is not applicable to this article.

## Abbreviations

The following abbreviations are used in this manuscript:

AI	Artificial intelligence
APC	Antigen-presenting cell
CAR	Chimeric Antigen Receptor
CAR-NK	Chimeric Antigen Receptor Natural Killer Cell
CAR-T	Chimeric Antigen Receptor T cell
CAF	Cancer-associated fibroblast
CDT	Chemodynamic therapy
CRISPR	Clustered regularly interspaced short palindromic repeats
CRS	Cytokine release syndrome
CT	Computed tomography
DC	Dendritic cell
ECM	Extracellular matrix
EPR	Enhanced permeability and retention
GMP	Good manufacturing practice
GSH	Glutathione
HCC	Hepatocellular carcinoma
ICI	Immune checkpoint inhibitor
ICANS	Immune effector cell–associated neurotoxicity syndrome
IL	Interleukin
LPHNP	Lipid–polymer hybrid nanoparticle
LNP	Lipid nanoparticle
MDSC	Myeloid-derived suppressor cell
MIL	Materials of Institute Lavoisier (metal–organic framework family)
MOF	Metal–organic framework
NIR	Near-infrared
NK	Natural Killer (cell)
NP	Nanoparticle
PDT	Photodynamic therapy
PEG	Polyethylene glycol
PSMA	Prostate-specific membrane antigen
RE-MOF	Rare-earth metal–organic framework
siRNA	Small interfering RNA
miRNA	microRNA
STING	Stimulator of interferon genes
TAM	Tumor-associated macrophage
TCR-T	T cell receptor–transduced T cell
TIL	Tumor-infiltrating lymphocyte
TLR	Toll-like-receptor
TME	Tumor microenvironment
RNPs	Ribonucleoprotein complexes
MDSCs	Myeloid-derived suppressor cells
ZnPP	Zinc protoporphyrin
